# Inflammatory Amplification: A Central Tenet of Uterine Transition for Labor

**DOI:** 10.3389/fcimb.2021.660983

**Published:** 2021-08-19

**Authors:** Kelycia B. Leimert, Wendy Xu, Magdalena M. Princ, Sylvain Chemtob, David M. Olson

**Affiliations:** ^1^Department of Obstetrics and Gynecology, University of Alberta, Edmonton, AB, Canada; ^2^Department of Pediatrics, Ophthalmology and Pharmacology, CHU Sainte-Justine Research Center, Montreal, QC, Canada

**Keywords:** uterine transition, inflammation, amplification, parturition, preterm birth, cytokines, IL-1β, IL-6

## Abstract

In preparation for delivery, the uterus transitions from actively maintaining quiescence during pregnancy to an active parturient state. This transition occurs as a result of the accumulation of pro-inflammatory signals which are amplified by positive feedback interactions involving paracrine and autocrine signaling at the level of each intrauterine cell and tissue. The amplification events occur in parallel until they reach a certain threshold, ‘tipping the scale’ and contributing to processes of uterine activation and functional progesterone withdrawal. The described signaling interactions all occur upstream from the presentation of clinical labor symptoms. In this review, we will: 1) describe the different physiological processes involved in uterine transition for each intrauterine tissue; 2) compare and contrast the current models of labor initiation; 3) introduce innovative models for measuring paracrine inflammatory interactions; and 4) discuss the therapeutic value in identifying and targeting key players in this crucial event for preterm birth.

## Introduction

The uterus is a dynamic organ that regularly undergoes cyclic changes and irregularly experiences considerable change in form and function. Throughout both the reproductive cycle and pregnancy, uterine modifications in cell proliferation, vascularization, and secretory outputs are induced by ovarian steroid hormones 17β-estradiol and progesterone to support menstruation, implantation, and decidualization (Bulletti et al., 1998; Gellersen and Brosens, 2003). The pregnant uterus increases more than 15-fold in size over the 40-week period of gestation to accommodate the growing fetus while actively maintaining a relaxed or quiescent state (Young, 2007). Then, as term approaches, the uterus must undergo extensive transition over a period of days into an active and contractile organ capable of performing the physiology of delivery. The process of uterine involution begins soon after birth and results in a return to the original uterine size and non-pregnant cycling state. Despite decades of obstetric research, progress in diagnosing risk for preterm birth and developing effective treatments has been disappointing, due in part to a lack of understanding of the physiology of birth and labor initiation. Therefore, although the uterus experiences considerable physiological and phenotypic change, the focus of this review article is on the extensive modification from the state of pregnancy to the state of labor or delivery. We call the sum total of genetic, cellular, and physiological changes that the uterus (and all maternal and fetal intrauterine tissues) must undergo during this period, *uterine transition*.

The question derives, how does a change as pervasive as pregnancy to parturition take place over such a short duration of time? The answer is amplification of the pro-inflammatory signals that activate the uterus for labor. These mediators are amplified through positive feedback interactions and cooperativity between ligands, cells, and tissues. A fundamental tenet of human physiology is the homeostatic balance in the body’s internal environment; the principle of negative feedback prevents uncontrolled increases of chemical and nervous signals. Consequently, uninhibited positive feedback interactions are predominantly observed in deregulated disease states, such as tumor growth in cancer (Singer et al., 2000; Menssen et al., 2012; Benesch et al., 2015), autoimmune/auto-inflammatory diseases (Beutler, 2009), and cellular senescence (Kandhaya-Pillai et al., 2017). Parturition uniquely forces the deviation of uterine physiology from normal homeostasis to ensure delivery, as maintenance of pregnancy past term compromises the health and ongoing viability of the mother and her fetus(es). Positive feedback mechanisms are not uncommon in reproductive and perinatal physiology. For instance, during ovulation estrogen drives the feedforward upregulation of gonadotropin-releasing hormone, which stimulates a surge in luteinizing hormone that results in the release of the mature oocyte (Holesh et al., 2021). Positive feedback or feedforward mechanisms achieve appropriate amplification to facilitate change at the cellular and tissue level over a short time period. The inflammatory system is an integral part of this upregulation process.

## Birth Is an Inflammatory Event

Many studies have established the role of inflammatory processes in both term and preterm birth. Human microarray studies demonstrate that the transition to labor is an inflammatory event not limited to a single gestational tissue (Haddad et al., 2006; Bollapragada et al., 2009; Mittal et al., 2010; Stephen et al., 2015; Migale et al., 2016; Sharp et al., 2016; Lui et al., 2018). In the myometrium, 471 genes were differentially expressed in preparation for term labor and 86% of the altered pathways were identified as inflammatory in nature (Mittal et al., 2010). Dominant gene changes in term laboring myometrium and cervix are involved in leukocyte movement, intercellular communication and cytokine signaling (Bollapragada et al., 2009). In another study, 1761 genes in the myometrium increased or decreased in preparation for term labor; a meta-analysis of publicly available transcriptomic data confirmed that laboring myometrium down-regulated muscle-specific processes while upregulating inflammatory processes (Sharp et al., 2016). In the choriodecidua, 796 genes increase or decrease in expression for term labor, with the activation of inflammatory or immune pathways leading the modifications to the transcriptome (Stephen et al., 2015). Moreover, six master regulators of transcriptomic changes for labor were identified by Ingenuity Pathway Analysis in choriodecidua and myometrium. Although the master regulators were different in choriodecidua from myometrium, downstream gene networks were similar and predominantly involved in inflammation (Lui et al., 2018). Amnion ‘activated’ for labor, characterized by high NFκB activity, exhibited an increase in the expression of 919 genes including pro-contractile and pro-inflammatory targets compared to non-activated amnion (Lim et al., 2012). Genes involved in neutrophil and monocyte recruitment were upregulated in term fetal membranes with labor, yet inflammatory gene expression in peripheral blood did not change (Haddad et al., 2006). Together, these studies affirm that uterine transition is a localized intrauterine inflammatory response.

In the majority of cases the inflammatory events of parturition occur without an infectious process (i.e. no indication of bacterial, viral or parasite invasion), termed *sterile inflammation* (Elliott et al., 2001; Romero et al., 2014; Nadeau-Vallée et al., 2016). In a study by Romero et al. (2014), the concentration of interleukin (IL)-6 in amniotic fluid was collected by amniocentesis and measured by immunoassay in a population of women who delivered preterm; the authors selected a concentration of IL-6 ≥ 2.6 ng/mL as the index for sterile intra-amniotic inflammation. When microbial footprints were identified in addition to IL-6 ≥ 2.6 ng/mL in amniotic fluid, cases were classified instead as microbial-associated intra-amniotic inflammation or infection. Inflammation was present in higher frequencies in deliveries at earlier gestational ages; 85% of women who delivered early preterm (<30 weeks of gestation) had intra-amniotic inflammation, with 60% experiencing sterile inflammation and 25% infection. When considering all women who delivered before 37 weeks of gestation, 26% experienced sterile inflammation and 11% infection. However, the group of women who did not show an indication for intra-uterine inflammation by these criteria had a mean time span of 4 weeks between the date of their amniocentesis and delivery (Romero et al., 2014). This is noteworthy as inflammatory amplification can occur rapidly over a period of days to weeks, so it is likely that the number of women experiencing sterile intra-amniotic inflammation may have been higher than these numbers demonstrate. A recent study combined untargeted mass spectrometry, proteomic technology, and single-cell mass cytometry immunoassay approaches to assess integrated trajectories in serial blood samples collected over the last 100 days of pregnancy. The authors identified a distinct molecular shift that occurred 2-4 weeks before delivery where pregnancy maintenance transitioned to pre-labor biology. The molecular shift involved metabolomic, proteomic, and immunologic systems and occurred with similar accuracy in both term and preterm women (Stelzer et al., 2021).

**Figure 1:**
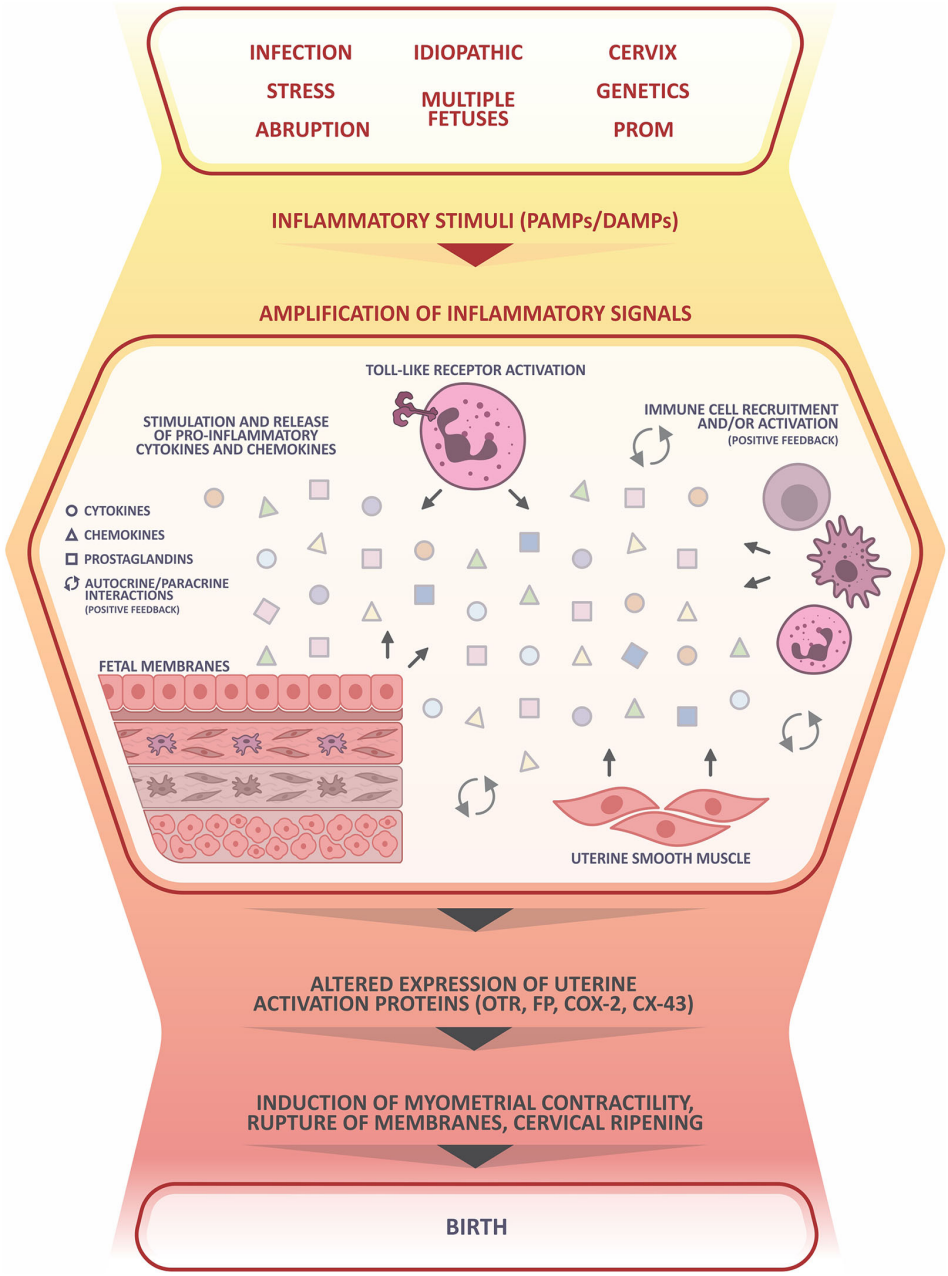
Inflammatory roadmap leading to birth and preterm birth. PAMPs or DAMPs are stimulated by infection, stress, or other initiators. They activate toll-like receptors (TLRs), which, in turn induce inflammasome assembly and stimulation of pro-inflammatory cytokines and chemokines. These pro-inflammatory agents stimulate leukocyte activation so that they infiltrate the uterus and release more cytokines and chemokines, thereby recruiting and activating more leukocytes in a positive feedback loop. These mediators also act on adjacent intrauterine tissues; in response, those tissues release additional inflammatory mediators. The amplification of inflammatory signals promotes altered expression of uterine activation proteins including COX-2, CX-43, FP, and OTR, among many others. Together, these changes comprise the transition of the uterus of pregnancy into the active and contractile uterus of delivery, stimulating the physiological events of delivery. PROM, premature rupture of membranes; PAMP, pathogen-associated molecular pattern; DAMP, damage-associated molecular pattern; OTR, oxytocin receptor; FP, Prostaglandin F_2α_ receptor; COX-2, cyclooxygenase-2; CX-43, connexin 43. Portions of this figure were created using BioRender.com.

In an infectious condition, the innate immune system is stimulated by pathogen-associated molecular patterns (PAMPs) produced by microorganisms. Amid a state of sterile inflammation, damage-associated molecular patterns (DAMPs) are produced in response to cellular stress, necrosis, and senescence. DAMPs are released by the increasingly physiologically stressed uterus, maturing fetus, and ageing placenta as parturition nears (Romero et al., 2014; Nadeau-Vallée et al., 2016). Fetal membrane senescence has been proposed as a fetal signal with an important role in labor initiation (Behnia et al., 2015). Senescent cells change their phenotype to a pro-inflammatory senescence-associated secretory phenotype but do not die, allowing for the continued release of markers of sterile inflammation that can drive positive feedback pathways involved in inflammation and senescence (Behnia et al., 2015). Both PAMPs and DAMPs activate toll-like receptors (TLRs), which induce inflammasome assembly, stimulation of pro-inflammatory cytokines and chemokines, and leukocyte activation, promoting the upregulation of uterine activation proteins (UAPs), prostaglandins, and their receptors (Cook et al., 2000; Cook et al., 2003; Ishiguro et al., 2016). Regardless of whether the initial stimulus is sterile (DAMP) or infectious (PAMP), a similar cascade of signaling events is involved, as depicted in **Figure 1**. The birth cascade is analogous to an iceberg - only a small fragment of the total picture is ‘visible’ at one time as the majority of upstream signaling processes are silent. By the time a woman presents with clinical symptoms of labor, considerable amplification has already taken place in the preceding days between upstream mediators. At this point, the birth cascade is nearly impossible to stop, similar to trying to intercept a train already travelling down the tracks at top speeds by standing on the tracks (Olson et al., 2008). This is why, despite substantial efforts, tocolytics have largely failed to inhibit preterm uterine contractions.

## Hormonal Regulation of Pregnancy

Unfortunately, a model organism that precisely depicts the physiology of human parturition does not exist. In addition to discrepancies in the type of placentation, gestational length, and number of offspring, considerable physiological differences exist in the hormonal control of parturition and labor initiation. Progesterone is a potent relaxant that inhibits uterine contraction during pregnancy, described as the ‘progesterone block’ theory by Csapo in 1956 (Csapo, 1956). In most animal species, labor occurs following the systemic withdrawal of progesterone (Murr et al., 1974; Sanyal, 1978; Mitchell and Taggart, 2009). Humans are fairly unique in that systemic levels of progesterone continue to rise throughout pregnancy and do not decrease until after the delivery of both fetus and placenta.

Birth timing in the non-human primate and guinea pig is independent of systemic progesterone withdrawal, hence these two animal models remain the most physiologically representative of pregnant women. However, non-human primates are a complex and expensive model requiring a large and experienced team, making them feasible at only a few centers in the world. The guinea pig model is more cost effective and pregnancy is maintained by placental progesterone production with a luteal-placental shift similar to humans. The review by Mitchell and Taggart describes the physiological mechanisms of parturition in each animal model in considerable detail (Mitchell and Taggart, 2009).

Even though progesterone withdrawal does not act as the ‘trigger’ for human labor, progesterone still has an essential role in the termination of pregnancy. RU-486 is a competitive progesterone antagonist which is able to induce birth at any point in gestation in every species including in women, conveying that pregnancy cannot continue in the absence of progesterone (Csapo, 1956; Frydman et al., 1988; Avrech et al., 1991; Dudley et al., 1996; Fang et al., 1997). Over the past two decades a model of functional progesterone withdrawal has emerged, demonstrating that in spite of an increase in serum progesterone levels, its effect upon pregnancy maintenance in women decreases before delivery (Mesiano et al., 2002). Research is still ongoing, but one possible mechanism is that transcription of the human nuclear progesterone receptor (*nPR*) gene is controlled by 2 different promoters, resulting in two nPR isoforms with different cellular and genomic actions: PR-A and PR-B (Kastner et al., 1990; Sartorius et al., 1994; Leonhardt et al., 2003). While PR-B promotes pro-gestational and anti-inflammatory transcription, PR-A inhibits PR-B activity as well as modulating its own pro-labor genomic actions (Tan et al., 2012; Nadeem et al., 2017; Peters et al., 2017). During pregnancy, ratios of PR-A to PR-B expression are low (~0.5). With advancing labor there is a shift in PR-A:PR-B nPR dominance and PR-A:PR-B ratios are much higher (~3) (Merlino et al., 2007). This balance in receptor isoforms also involves inflammation. Myometrial cells manipulated to express a low (~0.5) PR-A:PR-B ratio representative of levels during pregnancy exhibited the decreased expression of inflammatory mediators, whereas cells with a high (~3) PR-A:PR-B ratio demonstrated increased pro-inflammatory expression levels (Tan et al., 2012). Fetal membranes do not express nuclear progesterone receptors, but do express progesterone receptor membrane component 1 and 2 (PGRMC1 and PGRMC2) (Merlino et al., 2009; Lozovyy et al., 2021). In fetal membranes, PGRMC1 was found to be significantly lower in pPROM deliveries (Feng et al., 2014), PGRMC2 was significantly lower in both term labor and pPROM fetal membranes, and both were downregulated *in vitro* in response to oxidative stress (Lozovyy et al., 2021). Contrarily, another study found PGRMC2 to be significantly upregulated in choriodecidua at both term and preterm labor (Shankar et al., 2010). More research is needed to differentiate the specific roles of different progesterone receptor types in functional progesterone withdrawal in maternal and fetal intrauterine tissues.

## Inflammatory Amplification and Labor Initiation

It is not a new concept that a communication system involving steroid hormones, inflammatory processes, and paracrine interactions plays a key role in regulating human labor and parturition (Olson et al., 1995). In their 2009 review, Mitchell and Taggart introduced a concept defined as the “Modular Accumulation of Physiological Systems” (MAPS) (Mitchell and Taggart, 2009). MAPS depicts the amassing of a series of distinct events upregulated in parallel to induce human labor in lieu of a single stimulus. The accumulation of these events eventually reaches a ‘critical mass’ endpoint that results in parturition. More recently, concepts have been introduced by Talati et al. (2017) and Keelan (2018), termed “inflammatory load” and “inflammatory burden”. These concepts describe a steady increase in pro-inflammatory signal abundance over the gestational period. They hypothesize that human parturition occurs when pro-inflammatory mediators are upregulated and amplified until their signals exceed a threshold level that stimulates functional progesterone withdrawal and the other components required for the uterus to transition to its activated state for labor (Talati et al., 2017; Keelan, 2018). Hence human parturition is governed by localized paracrine interactions involving pro-inflammatory and pro-contractile systems. At term these intertwined systems drive amplification towards a critical and irreversible threshold level that becomes the trigger for human parturition.

As inflammation has a central role in all of the aforementioned mechanisms, there is value in investigating more specifically its role in the most commonly used *in vivo* parturition mouse models: spontaneous term labor (sTL), progesterone receptor antagonist RU486-induced preterm birth (PTB), and gram-negative endotoxin lipopolysaccharide (LPS)-induced PTB. Studies from two different research groups have compared gene or protein expression profiles in these three different models through labor and delivery. Both groups determined that while sTL and RU486-PTB groups display similar profiles, the LPS-PTB mouse group profile is distinct (Shynlova et al., 2013; Migale et al., 2016). Shynlova et al. (2013) established the differences between groups by examining the expression of a series of inflammatory and uterine activation genes *via* RT-PCR, cytokine and chemokine outputs *via* multiplex analysis, and analyzing the populations of immune cells infiltrating into the myometrium. While expression levels in all three groups were similar postpartum, the LPS-PTB group differed before and at labor with a much more robust pro-inflammatory response and no measured increase in uterine activation protein (UAP) Connexin-43 (Shynlova et al., 2013). Migale et al. (2016) instead employed uterine temporal transcriptomics using multivariate modeling approaches to compare the three models. The principal component analysis identified that the LPS-PTB group demonstrated a divergent trajectory over time from the sTL and RU486-PTB groups. While changes to the transcriptome in sTL and RU486-PTB groups were 60% similar and did involve some degree of pro-inflammatory upregulation, 80% of the gene changes in the LPS-PTB group were unique to that group alone and were predominantly involved in inflammation (Migale et al., 2016). Both research groups suggested that the RU486-PTB group was most similar to sTL as progesterone withdrawal is a fundamental component of labor initiation in mice, whereas inflammation may instead be involved in uterine remodeling postpartum and not labor initiation and delivery itself (Shynlova et al., 2013; Migale et al., 2016). Uterine remodeling postpartum or uterine involution is characterized by a reduction in the size and vascularization of the uterus so that it may return to a non-pregnant state of cycling.

These findings align with an earlier microarray study that compared uterine gene expression in spontaneous term delivery between mouse and human (Bethin et al., 2003). While genes involved in immune/inflammatory regulation, protease/protease inhibitors, and cell adhesion categories were downregulated for labor in the mouse uterus, they were instead upregulated in human myometrium, suggesting a lesser contribution of progesterone withdrawal and a more abundant contribution of inflammation in human parturition (Bethin et al., 2003). Other studies have supported that inflammation is not critical to mouse labor and delivery by demonstrating that depleting specific subsets of circulating immune cells such as neutrophils (Timmons and Mahendroo, 2006; Rinaldi et al., 2014), mast cells (Menzies et al., 2012), and total polymorphonuclear leukocytes (including neutrophils, eosinophils, and basophils) (Filipovich et al., 2015) had no effect on birth timing. Collectively, these data suggest that in mice there is an involvement of inflammatory processes in labor, but unlike progesterone withdrawal, these processes may be dispensable. However, a recent PTB mouse model demonstrated that inflammation can induce preterm delivery without a fall in systemic progesterone. The authors collected exosomes on day 18 of gestation and injected the exosomes into day 15 pregnant mice. The exosomes migrate into maternal cervix, myometrium, and fetal membranes and induced a localized inflammatory response *via* paracrine mechanisms that resulted in preterm delivery in 80% of mice, even while systemic progesterone levels remained constant (Sheller-Miller et al., 2019). Further research on labor initiation is needed, but it seems that parturition is a synchrony of multiple mechanisms and redundancy assures delivery in the absence of the other triggers.

The question going forward is, are inflammatory mediators in the human uterus relevant for only uterine involution or are they also relevant for uterine activation and expulsion? In the same study, Migale et al. (2016) then compared the gene profiles of the three mouse birth cohorts to human uterine transcriptome data collected before and after spontaneous term labor. Interestingly, of the three mouse cohorts, the gene profile of the human data set most closely paralleled the LPS-PTB model (Migale et al., 2016). Undoubtedly, the LPS-PTB model is not without its limitations, as it models systemic inflammation and not the localized intrauterine inflammation exhibited in human parturition. However, the comparison endorses the intrinsic role of inflammation in human parturition. A 2005 study used directed graphs of causal influence matched to a human RT-PCR data set to statistically compare the likelihoods of different physiological scenarios for the initiation of human labor onset. The novel computational approach identified inflammatory processes as the predominant stimulus for human labor onset with high probability (Bisits et al., 2005). The Mesiano group has proposed tissue-level inflammation as a mechanism for the antagonism of progesterone-mediated gene changes that achieve functional progesterone withdrawal. IL-1β and prostaglandin (PG)F_2α_, working through their receptors IL-1R and FP, increase the PR-A/PR-B ratio of progesterone receptor isoforms and increase the abundance and stability of PR-A (Madsen et al., 2004; Amini et al., 2016; Peters et al., 2017), thereby promoting pro-inflammatory expression and pro-labor transitioning (Tan et al., 2012). These findings emphasize a need for developing more representative methods for examining the molecular events of human parturition while expressing caution when interpreting findings from *in vivo* parturition animal models.

## Uterine Transition Involves More Than Just the Myometrium

To properly discuss the molecular changes involved in uterine transition and understand how paracrine signaling establishes localized changes for labor, the physiology of each uterine tissue must be considered separately and in cooperation with neighboring tissues. Within the pregnant uterus, signaling occurs between maternal and fetal gestational tissues. The coordination required to effect the five separate yet physiologically interrelated events of parturition: membrane rupture, cervical dilatation, myometrial contractility, placental separation and uterine involution (Olson et al., 1995), involves considerable tissue interactions.

### Myometrium

The myometrium, or uterine smooth muscle, is predominantly comprised of fusiform-shaped cells. For the myometrium to contract as a coordinated unit during labor, electrical coupling between individual smooth muscle cells is essential. Ions and small molecules flow from cell to cell through gap junctions formed by groups of connexin proteins. These gap junctions allow intercellular communication and propagation of action potentials between smooth muscle cells, enabling contraction of a synchronized uterine musculature through excitation-contraction coupling (Garfield et al., 1977; Sims et al., 1982). Connexin proteins, such as Connexin (CX) 43, increase in expression at labor and are stimulated by rising levels of estrogens and progesterone *via* the PR-A receptor (Garfield et al., 1980; Chow and Lye, 1994; Di et al., 2001; Nadeem et al., 2017). In more than 80% of mice with a deletion of smooth muscle-specific *Cx43*, labor is delayed, likely due to the impaired electrical coupling of the myometrium (Döring et al., 2006). In addition to enhanced electrical signal transmission throughout the myometrium, the sensitivity of the uterus to contractile agonists is also increased at term. This development occurs as a result of the altered expression of a series of uterine activation proteins (UAPs), which change in expression for labor to increase systems of uterine contractility or suppress uterine relaxation (Cook et al., 2000). These markers of activation include connexins, cyclooxygenases, receptors and agonists of contractile stimulators, as presented in **Table 1**.

**Table 1:** Uterine activation proteins change their expression for labor*.

Uterine activation protein	Role	Change for labor	Key citations
Cyclooxygenase 2 (COX-2)	Enzyme catalyzing the rate limiting step in prostaglandin synthesis	↑	(Hirst et al., 1995; Slater et al., 1995; Mijovic et al., 1997; Hirst et al., 1998; Cook et al., 2000)
Prostaglandin (PG)F_2α_ and PGF_2α_ receptor (FP)	Uterotonic G-protein coupled receptor (GPCR) and agonist	↑	(Skinner and Challis, 1985; Romero et al., 1996; Brodt-Eppley and Myatt, 1999; Cook et al., 2000; Al-Matubsi et al., 2001)
Oxytocin (OT) and OT receptor (OTR)	Uterotonic G-protein coupled receptor (GPCR) and agonist	↑	(Fuchs et al., 1982; Fuchs et al., 1984; Chibbar et al., 1993; Fang et al., 1996)
Connexin 43 (CX-43)	Gap junction protein essential for myometrial connectivity	↑	(Garfield et al., 1977; Lye et al., 1993)
Endothelin 1 (ET-1) and ET-1 receptor ET_A_	Vasoconstrictor, uterotonic agonist and G-protein coupled receptor (GPCR)	↑	(Kozuka et al., 1989; Word et al., 1992; Yallampalli and Garfield, 1994; Arthur et al., 2008)
Inducible nitric oxide synthase (iNOS)	Enzyme catalyzing production of nitric oxide (NO)	↓	(Ali et al., 1997; Bansal et al., 1997)
NmUR2	Neuropeptide, uterotonic agonist	↑	(Nadeau-Vallée et al., 2016)

*This is just a small representation of hundreds of proteins that increase or decrease their expression in the process of uterine activation for labor.

The shift in UAP expression profiles at labor is regulated by pro-inflammatory mediators, such as Interleukin-1β (IL-1β) and IL-6. Many markers of uterine activation have binding sites for cytokine-induced transcription factors in their promoter regions. For example, FP’s promoter region contains binding sites for both nuclear factor (NF)κB and NFIL-6, supporting more than one route for cytokine-mediated transcriptional regulation (Zaragoza et al., 2004; Zaragoza et al., 2006). In addition to FP, pro-inflammatory cytokines have been shown to regulate expression of COX-2 (Molnár et al., 1993; Kniss et al., 1997; Belt et al., 1999; Rauk and Chiao, 2000; Hirsch et al., 2006), OTR (Fang et al., 2000; Rauk and Friebe-Hoffmann, 2000; Schmid et al., 2001), CX-43 (Tonon and D’Andrea, 2000), ET-1 (Woods et al., 1999; Molet et al., 2000), iNOS (Marczin et al., 1993), PGs (Mitchell et al., 1990; Mitchell et al., 1991; Pollard et al., 1993), and NmUR2 (Nadeau-Vallée et al., 2016).

Steroid hormones estrogen and progesterone are also important regulators of UAPs. Progesterone acts through its PR-B receptor to suppress UAP expression through the modulation of transcriptional activity, such as antagonizing NFκB *via* NFκB inhibitor alpha (IκBα) (Loudon et al., 2003; Hardy et al., 2006). The pro-pregnancy actions of progesterone can be amplified by the activation of the cAMP pathway, which is also thought to play a role in maintaining uterine quiescence (Amini et al., 2019). In addition, progesterone represses OTR through direct binding of the receptor, subsequently preventing production of inositol phosphate and mobilization of calcium ions (Ca^2+^) (Grazzini et al., 1998; Dunlap and Stormshak, 2004). Estrogen has the contrary effect, and promotes UAP expression *via* estrogen response elements in UAP promoter regions (Garfield et al., 1980; Soloff et al., 1983; Chibbar et al., 1995; Lefebvre et al., 1995; Di et al., 2001). Estrogen can also interact with OTR through extranuclear pathways to enhance myometrial contractility (Maiti et al., 2011; Welsh et al., 2012). Oxytocin stimulation, *via* OTR, induced activation of NFκB and upregulated gene expression of IL-6, IL-8, CCL5 and COX-2 in myometrial cells (Kim et al., 2015). UAPs can also be modulated by the stretching of the uterus to accommodate the growth and development of the fetus. The mechanical stretch of cultured human myometrial cells, amniocytes, fetal membranes, and decidua stimulates the induction of a series of pro-inflammatory cytokines and chemokines (Maehara et al., 1996; Loudon et al., 2004; Kendal-Wright, 2007; Hua et al., 2012; Adams Waldorf et al., 2015; Lee et al., 2015), upregulates leukocyte recruitment (Lee et al., 2015) and upregulates COX-2 and OTR in myometrial cells (Sooranna et al., 2004; Terzidou et al., 2005). The inflation of an intrauterine balloon (150 mL) in the uterus of non-laboring women at term resulted in the increased synthesis of prostaglandins and labor induction (Manabe et al., 1982). Similarly, in non-human primates inflation of an intraamniotic balloon resulted in a spike in cytokine and prostaglandin release, followed by preterm delivery in 3 of the 6 animals (Adams Waldorf et al., 2015). Uterine stretch increased CX-43 and OTR in the rat (Ou et al., 1997; Ou et al., 1998), and OTR and COX-2 in the sheep (Wu et al., 1999).

### Cervix

The cervix is the lower section or ‘neck’ of the uterus, and comprises columnar mucous-producing glandular epithelial cells, squamous epithelial cells, cervical fibroblasts and smooth muscle cells. However, the predominant composition of the cervix is importantly extracellular matrix, containing collagen, elastin, and glycoproteins. As the uterus grows and stretches during pregnancy to accommodate the growing fetus, the cervix has the important role of ‘uterine gatekeeper,’ maintaining rigidity despite increasing tension, pressure, and weight. The high collagen content of the cervix assists in this feat of physical integrity.

As birth approaches, the cervix must transition from closed and rigid to dilated and soft, stretching to accommodate the passage of the fetus through the birth canal. This transition occurs in two stages: cervical softening and ripening. Softening occurs gradually through gestation beginning near the end of the first trimester, whereas ripening is a more accelerated process occurring over the last days to weeks of pregnancy resulting in the loss of cervical integrity (Timmons et al., 2010). Collagen is remodeled by the re-organization of the matrix structure and through increasing levels of collagenases (Granström et al., 1992; Osmers et al., 1992; Shi et al., 1999). Glycosaminoglycans (GAGs) increase through pregnancy, rearranging collagen for increased cervical distensibility (Osmers et al., 1993). Mature collagen crosslink density decreases over the course of pregnancy (Yoshida et al., 2014), and collagen is degraded by increasing levels of collagenases and matrix metalloproteinases (MMPs), with only 30% of non-pregnant collagen levels remaining at term (Uldbjerg et al., 1983). Cervical ripening is influenced by steroid hormones, prostaglandins, growth factors and invading leukocytes (Stjernholm et al., 1996; Gu et al., 2012; Payne et al., 2012; Blesson et al., 2013; Kirby et al., 2016). First proposed to be an inflammatory process by Liggins in 1981, leukocytes and their products are involved in mediating changes to the cervical extracellular matrix (Junqueira et al., 1980; Liggins, 1981; Osmers et al., 1992; Sennström et al., 2000; Osman et al., 2003).

### Decidua

The non-pregnant uterus is lined by endometrium, consisting of endometrial stromal cells, which differentiate into decidual cells through the process of decidualization. Once the blastocyst successfully implants, the decidua completely envelops the blastocyst and continues to grow and foster the invasion of trophoblasts and support placentation. Uterine natural killer cells, decidual macrophages, uterine dendritic cells, and regulatory T (T_reg_) cells are present in decidua and involved in vascular remodeling and adaptive immune roles suppressing anti-embryo maternal immune responses (Hanna et al., 2006; Seavey and Mosmann, 2008; Gomez-Lopez et al., 2010; Mori et al., 2016). The decidua is essential not only in the establishment of pregnancy, but to maintain and support pregnancy throughout gestation through the production of important hormones and growth factors.

As labor approaches, the physiology of the decidua changes. Like the myometrium, the decidua upregulates UAPs and undergoes activation. COX-2 is considerably elevated in the decidua in late gestation and labor which makes it a major producer of PGF_2α_ (Hirst et al., 1998), and uterotonic OTR and FP receptors are expressed in decidua (Okazaki et al., 1981; Fuchs et al., 1982). Decidual FP expression levels increase four-fold with labor, while expression of all progesterone receptor (PR) isoforms diminish (Goldman et al., 2005; Makino et al., 2007). In addition, pro-inflammatory mediators such as IL-6, CCL2, CCL4, CCL5, CXCL8, and CXCL10 are upregulated in the decidua at term and correlate with decidual leukocyte abundance (Osman et al., 2003; Hamilton et al., 2013; El-Azzamy et al., 2017). Leukocyte infiltration and activation in the decidua precedes term and preterm delivery in both human and rat (Hamilton et al., 2012; Xu et al., 2016). *Ex vivo* organ culture of decidua stimulated with PGF_2α_ upregulated expression of MMP-2 and -9, enzymes involved in the degradation of adjoining fetal membranes, while decreasing expression of the tissue inhibitor of metalloproteinases-1 (TIMP-1) (Ulug et al., 2001). The mechanical stretch of decidual cells increased activity of MMP-1 while also upregulating CXCL1 and CXCL8 mRNA and protein (Zhao et al., 2013).

### Fetal Tissues

The human fetal membranes (hFM), consisting of chorion and amnion, envelope and protect the fetus throughout pregnancy. Chorionic trophoblasts invade the maternal decidua and form a highly integrated choriodecidua maternal-fetal interface (Mitchell and Powell, 1984). By mid-pregnancy, the fetal membranes line the entire uterine cavity. As the growing fetus kicks, stretches, and moves, the fetal membranes maintain viscoelasticity to withstand movements and avoid rupture, safely containing the fetus and surrounding amniotic fluid (Parry-Jones and Priya, 1976; Lavery and Miller, 1979; Patrick et al., 1982). This characteristic is modified in preparation for labor, as the membranes must then rupture to permit passage of the fetus through the birth canal. Membrane rupture is facilitated by biomechanical and biochemical changes weakening the amnion and chorion combined with the mechanical force of uterine contractions at labor (Kumar et al., 2011; Puthiyachirakkal et al., 2013). The amnion is the load-bearing structure in the fetal membranes; although the tissue is very thin, mechanical testing has demonstrated that it is stiffer, tougher and stronger than the chorion, and the strength of the amnion decreases significantly in preparation for labor (Helmig et al., 1993; Bircher et al., 2019). The amnion and chorion layers are connected by extracellular matrix with a high collagen content, this matrix must also undergo enzymatic breakdown during membrane rupture.

Increasing amounts of oxidative stress and cell senescence occur through the gestational period, prompting the release of sterile inflammatory mediators of parturition, including DAMPs and mediators of the senescence-associated secretory phenotype (SASP) (Menon et al., 2017). Fetal membranes overlaying the cervix have been shown to express less antioxidant enzyme activity than the surrounding areas of the membrane, making that zone more susceptible to oxidative stress, damage, and rupture (Chai et al., 2012). Fetal membrane senescence has been proposed to function as a biological clock, as senescence can drive inflammatory upregulation and contribute to birth timing and membrane rupture (Menon et al., 2016). The inflammasome, responsible for IL-1β production, is activated at term labor in both the fetal membranes and in choriodecidual leukocytes (Gomez-Lopez et al., 2017; Romero et al., 2018). Inflammatory mediators induce MMPs, which remodel extracellular matrix through the degradation of collagen. For example, *in vitro* stimulation of TNL fetal membrane explants with TNFα and IL-1β induced membrane weakening, apoptosis, and collagen remodeling, as well as the upregulation of MMP-9 and downregulation of TIMP-3 (Kumar et al., 2006). Thrombin directly induces weakening of the amnion membrane *in vitro*, whereas TNFα and IL-1β instead act indirectly on the amnion by stimulating the release of intermediates from the choriodecidua (Kumar et al., 2011; Puthiyachirakkal et al., 2013). MMP-2 and MMP-9 degrade collagen IV, a prominent collagen in the basement membrane of the amnion, in chorionic cytotrophoblasts and in connective tissue (Malak et al., 1993; McLaren et al., 2000).

### Maternal Leukocytes

In preparation for labor, the human fetal membranes exhibit chemotactic properties, releasing chemoattractant that induces the migration of maternal peripheral leukocytes into intrauterine tissues (Gomez-Lopez et al., 2009; Gomez-Lopez et al., 2011; Takeda et al., 2017). Concurrently, the abundance of granulocytes, monocytes, and lymphocytes increase and circulating leukocytes become more responsive to chemoattractant by upregulating key receptors and intracellular signaling molecules (Yuan et al., 2009; Zhang et al., 2017; Lee et al., 2020). Pro-inflammatory cytokine and chemokine abundance in human intrauterine tissues and amniotic fluid increases at labor (Halgunset et al., 1994; Elliott et al., 2001; Osman et al., 2003), and leukocyte infiltration into myometrium (Thomson et al., 1999), decidua (Xu et al., 2015; Arenas-Hernandez et al., 2019), cervix (Bokström et al., 1997; Osman et al., 2003) and fetal membranes (Gomez-Lopez et al., 2009; Jacobs et al., 2021) has been characterized at term. Leukocyte extravasation into gestational tissues provides another level of feed-forward tissue-level inflammatory amplification as the leukocytes interact with surrounding cells, as demonstrated in **Figure 1**. Mediators released by the infiltrated leukocytes contribute to the transitional events described above such as membrane rupture and cervical ripening, as well as the preparation for tissue repair and remodeling after delivery (Gomez-Lopez et al., 2010).

## Inflammatory Cooperativity Between Ligands and Tissues

Nearly all we know about parturition in humans is derived from *in vitro* studies examining each mediator in isolation or *ex vivo* studies in individual tissues. However, as described throughout this review, the *in vivo* environment is much more complex and paracrine interactions between cells and adjacent tissues are an important component of that complexity. We must pursue other avenues to investigate parturition biology and create useful models for collecting the crucial pre-clinical data needed to develop clinical products for women’s pregnancy health. Studies in recent years have shown that there is a lot we can learn from studying ligands in combination and/or building more precise models to better mimic the *in vivo* situation.

For example, two of the most important mediators involved in the transcriptional regulation of IL-6 are IL-1β and TNFα, as both cytokines induce the transcription factors activator protein (AP)-1, nuclear factor (NF)κB, and NFIL-6 (Akira et al., 1990; Karin, 1995; Khanjani et al., 2011). These transcription factors can interact in complexes to cooperatively regulate IL-6 transcription. NFIL-6 and NFκB together induced a synergistic 40-fold increase in IL-6, 20 times higher than their individual effects (Matsusaka et al., 1993). Collectively, AP-1, NFκB, and NFIL-6 formed a transcription factor complex that resulted in a nearly 300-fold increase in IL-6, much greater than the outcome of individual or paired combinations (Faggioli et al., 2004). In astrocytes, the transcription factors CREB, NFκB, and CBP form a complex that synergistically upregulates transcription of both IL-6 and COX-2 (Spooren et al., 2010). CCL2 synergizes with a series of different chemokines, including IL-8 and CXCL6, to induce amplified leukocyte chemotaxis (Gouwy et al., 2004; Gijsbers et al., 2005). This concept is not new to immunology, as amplification is employed to rapidly mobilize leukocytes in immune defense responses. The role of cooperative regulation must be investigated further as part of the signaling contributing to uterine transition, especially as many UAPs have binding sites in their promoter regions for several inflammatory transcription factors. In human myometrial cells, sequential combinations of IL-1β and PGF_2α_ upregulated cytokine IL-6 and UAP COX-2 to levels much higher than the sum of each mediator’s individual effects (Leimert et al., 2019).

Likewise, scientific models must be designed to study interactions between adjacent cell and tissue types within the maternal-fetal interface. In recent years, a number of studies have demonstrated paracrine interactions through the development of different intrauterine co-culture models. Uterine myocytes co-cultured with primary term non-laboring monocytes released much greater levels of IL-6 and IL-8 than the output of each cell type individually. Furthermore, the myocyte-monocyte crosstalk also elevated uterine myocyte contractility (Rajagopal et al., 2015). In another study, myometrial cells stimulated with CRH and co-cultured with THP-1 monocytes upregulated higher outputs of PGE_2_, cytokines and chemokines, and UAP expression compared to myometrial cells cultured alone (You et al., 2014). In addition, myometrial cells co-cultured with differentiated macrophages upregulated myocyte contractility and CX-43 protein expression following LPS stimulation (Wendremaire et al., 2020). Lee et al. (2015) treated uterine endothelial cells with conditioned medium collected from cultures of uterine myocytes that had been stretched *in vitro* (Lee et al., 2015). As a result, the endothelial cells exhibited an upregulation in cell adhesion molecules on their cell surface. Moreover, leukocytes exposed to the myocyte-conditioned medium upregulated monocyte surface markers of activation, increased granulocyte binding to uterine endothelial cells, and upregulated transendothelial migration (Lee et al., 2015). A follow-up study demonstrated that even without stretch, conditioned medium collected from cultured primary term myometrial cells or primary term decidual cells induced an upregulation of activation markers involved in adhesion and migration in early pregnant peripheral leukocytes, supporting a role for paracrine interactions between cell types in inducing leukocyte extravasation into gestational tissues (Farine et al., 2017). To model the paracrine delivery of inflammatory signals from fetal to maternal tissues *in vitro*, exosomes were collected from primary amnion epithelial cells and administered to cultures of myometrial cells or primary decidual cells. Over 24 hours, exosomes entered the maternal cells and induced inflammatory upregulation, significantly increasing IL-6, IL-8, PGE_2_ and NFκB (Hadley et al., 2018).

Our group co-cultured primary uterine myocytes with fetal membrane tissue explants and found that 18 inflammatory mediators were synergistically upregulated when compared to the tissues cultured in isolation (Leimert et al., 2018). Interestingly, the cytokines and chemokines with the highest total concentration output or largest fold-change increase in this co-culture model resembled the lists of mediators identified as the most altered gene pathways in preparation for labor in the human microarray studies previously discussed (Haddad et al., 2006; Bollapragada et al., 2009; Mittal et al., 2010; Stephen et al., 2015), supporting the validity of this method in modeling pro-inflammatory amplification in uterine transition for labor. Keelan et al. (2009) developed an *ex vivo* model using an Ussing chamber that allows for the independent stimulation of the maternal (decidual) and fetal sides of the human fetal membranes. Using this model, it is possible to measure separate secretory outputs from each side as well as study the barrier and transfer functions of the tissue. The authors demonstrated that when the maternal decidual side is stimulated with LPS, cytokines are significantly upregulated on both the maternal and fetal sides, even though LPS does not pass through to the fetal compartment. The authors suggested that the inflammatory upregulation in the fetal compartment likely occurred due to a cascade of paracrine interactions taking place throughout the layers of the tissues (Keelan et al., 2009). More recently, this concept has been developed further. The fetal membrane organ-on-chip, a novel *in vitro* system, utilizes a multi-chamber microfluidic device to study interactions between fetal amnion epithelial cells and maternal decidual stromal cells with more control, higher sensitivity, and increased precision (Richardson et al., 2019). Further optimization has resulted in a microfluidic organ-on-chip system of the feto-maternal interface, containing primary decidual, chorion, and amnion (mesenchyme and epithelial) cells. Using this system, the authors were able to model the propagation of ascending maternal infection and show cell type specific immune responses to the exposure. LPS treatment of decidual cells resulted in inflammatory upregulation in decidual cells within 24 hours, fetal chorion within 48 hours, and fetal amnion cells within 72 hours (Richardson et al., 2020). A cervical epithelial organ-on-chip system has also been recently developed, incorporating endocervical and ectocervical epithelial cells (Tantengco et al., 2021). These creative model systems offer alternative methodologies for research questions that may not be answered precisely with accessible animal models.

## Key Upstream Mediators in Uterine Transition

Characterizing the central mediators within the pro-inflammatory network of uterine transition is of the utmost importance. Two mediators intrinsically involved in the tissue-level transformation of maternal and fetal intrauterine tissues for labor are IL-1β and IL-6. IL-1β is a member of the IL-1 cytokine family, generated following the activation of pattern recognition receptors such as TLRs and NOD-like receptors. IL-1β signals through a heterodimeric complex; the functional ligand binding chain IL-1R1 binds IL-1 and complexes with its accessory protein (IL-1RAcP or IL-1RAcPb) to stimulate a downstream cascade that results in the activation of transcription factors AP-1 and NFκB. IL-1 is a potent cytokine, able to induce sizable biological responses at low concentrations. A maximum cellular response to IL-1 occurs at only 1% occupancy of the IL-1R1 receptors (Dower and Sims, 1990). IL-1 activity must therefore be highly regulated through negative feedback; the endogenous IL-1R antagonist (IL-1ra) competes with IL-1 ligand to bind and sequester IL-1R1 (Seckinger et al., 1987). High levels of IL-1ra in both secretory and intracellular forms are expressed endogenously; bioavailability of IL-1ra ligand is typically several thousand times higher than IL-1 ligand (Arend, 1993). The second IL-1 receptor, IL-1R2, also binds IL-1 ligand and IL-1RAcP but is lacking a TIR domain, thereby scavenging IL-1 and functioning as a decoy receptor without the production of signal transduction (Boraschi and Tagliabue, 2013). In addition to IL-1R2, soluble forms of IL-1R1 and IL-1RAcP also exist as decoy receptors to sequester IL-1 ligand without signal transduction or cellular response.

At term labor, IL-1β is upregulated in amniotic fluid (Romero et al., 1990; Romero et al., 1992) and intrauterine tissues including myometrium, decidua, cervix and fetal membranes (Ammälä et al., 1997; Elliott et al., 2001; Osman et al., 2003). The endogenous antagonist IL-1ra decreases in cervicovaginal fluid at spontaneous term labor onset (Heng et al., 2014). IL-1β has an influential coordinating role in the regulation of pro-inflammatory and pro-labor genes in intrauterine tissues. In decidual cells, microarray data presented a significant upregulation of 350 transcripts and the activation of an estimated 57 transcription factors following IL-1β stimulation (Ibrahim et al., 2016). A short exposure to IL-1β upregulates 98 inflammatory genes in PHM1-41 uterine myocytes by at least 3-fold, including COX-2 (7.9-fold), NFκB (10.9-fold) and genes involved in extracellular matrix (ECM) remodeling, cell adhesion and angiogenesis (Chevillard et al., 2007).

IL-1β is a potent stimulator of prostaglandin synthesis in all gestational tissues *via* COX-2 induction (Romero et al., 1989; Brown et al., 1998; Bartlett et al., 1999; Rauk and Chiao, 2000; Ibrahim et al., 2016), and contributes to functional progesterone withdrawal by increasing the stability and abundance of the PR-A receptor (Amini et al., 2016; Peters et al., 2017). The excitability of primary human myometrial cells is upregulated by IL-1β, increasing both basal calcium entry and spontaneous calcium transients (Tribe et al., 2003). RU486-induced rat deliveries demonstrated increased uterine expression of IL-1R1, IL-1R accessory proteins AcP and AcPb, and decreased expression of decoy receptor IL-1R2 (Ishiguro et al., 2016). Mice administered an intrauterine injection of IL-1β exhibited a significant increase in mRNA abundance of genes encoding for COX-2, FP, OTR, CX-43, MMP-1, 3, 9, IL-1β, IL-6, IL-8, CCL2 and IL-1R1 in the myometrium compared to vehicle-injected animals (Nadeau-Vallée et al., 2015). Human myometrial cells stimulated with IL-1β also upregulate mRNA abundance of the IL-1 receptors and accessory proteins (Leimert et al., 2019), further contributing to inflammatory amplification by minimizing negative regulation of IL-1β. It is therefore not surprising that IL-1β injection effectively induces preterm birth in a range of animal models, such as the mouse (Romero et al., 1991; Yoshimura and Hirsch, 2005; Nadeau-Vallée et al., 2015), the rabbit (in combination with TNFα) (Bry and Hallman, 1993), and the nonhuman primate (Sadowsky et al., 2006).

One of the key mediators stimulated by IL-1β is IL-6, a cytokine involved in a wide range of biological functions, including implantation, pregnancy, and parturition. IL-6 is produced in abundance by the myometrium, cervix, and choriodecidua before and during labor (Osman et al., 2003), and elevated or deficient IL-6 levels are associated with infertility, fetal loss, and other pregnancy disorders (Prins et al., 2012). IL-6 is measured in amniotic fluid in low concentrations beginning in the second trimester, but at term, concentrations of IL-6 increase from 399 to 4800 pg/mL with labor onset (Opsjłn et al., 1993). Human amnion and decidual cells stimulated by physiological concentrations of IL-6 (as measured in amniotic fluid at term) upregulate the production of prostaglandins (Mitchell et al., 1991). In the rat, IL-6 increases expression of uterotonic receptor OTR in the uterus (Fang et al., 2000). Mice with a null mutation for *Il6* (*Il6-/-)* deliver 24 hours later than mice with an IL-6 presence due to a 24-hour delay in the upregulation of UAPs, and normal birth timing can be restored with exogenous IL-6 administration (Robertson et al., 2010). However, although *Il6-/-* mice deliver late and IL-6 levels are upregulated in LPS-induced preterm birth mouse models (Nadeau-Vallée et al., 2015), IL-6 injection alone is not sufficient to induce preterm labor (Yoshimura and Hirsch, 2003) and IL-6 does not stimulate uterine contractions (Dajani et al., 1994). Nonetheless, IL-6 has been pursued as a diagnostic marker for preterm birth. Women who experienced spontaneous preterm births before 35 weeks had significantly elevated IL-6 levels in amniotic fluid in second trimester (Wenstrom et al., 1998) and in cervical fluid at 24 weeks of gestation (Goepfert et al., 2001). In addition, Robertson’s group demonstrated the importance of IL-6 in mediating T-cell population changes in late pregnancy in the mouse using an *Il6-/-* model. IL-6 mediates CD4^+^ T cell differentiation into Th17 cells and CD8^+^ T cell differentiation into Foxp3^+^ T regulatory cells, while decreasing the number of decidual Th9 cells (Gomez-Lopez et al., 2016). These IL-6-mediated T-cell population changes contribute to the progression of parturition. Exogenous IL-6 administration restored T cell populations by 60% and recovered birth timing (Gomez-Lopez et al., 2016).

## Inflammatory Amplification Propels the Gears

**Figure 2:**
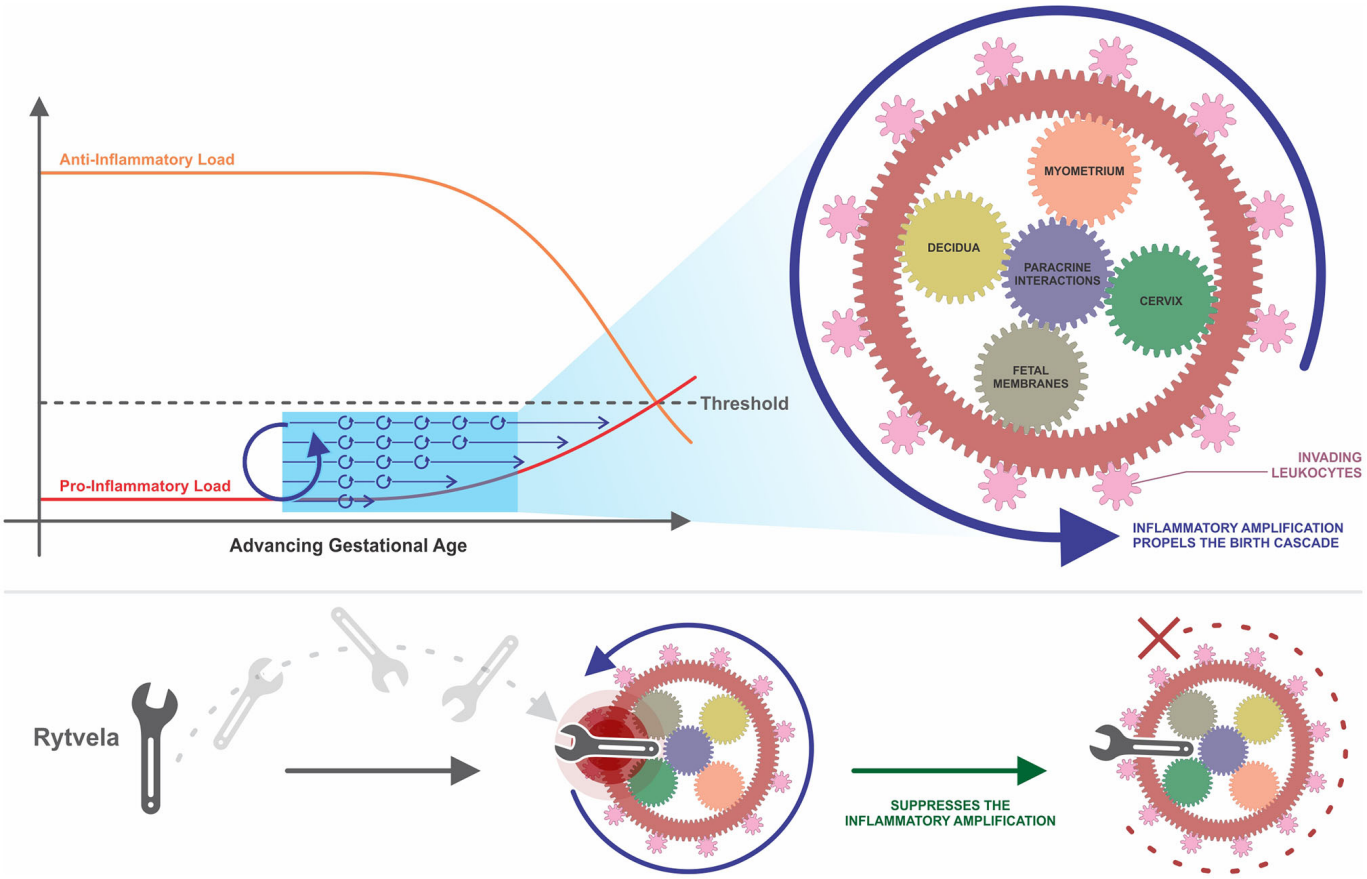
A conceptual framework of uterine transition. This framework incorporates the concepts of MAPS and anti- and pro-inflammatory load introduced by Mitchell and Taggart (2009), Talati et al. (2017), and Keelan (2018). Parallel up-regulatory events accumulate over time, driven by paracrine interactions between various cells and tissues in the intrauterine environment. Amplification occurs within and between these pathways, which begins to propel the momentum of the larger wheel. The pro-inflammatory load continues to build while the anti-inflammatory load, such as the progesterone blockade, diminishes. These two loads ultimately approach each other, creating a threshold value that once crossed, triggers irreversible labor and parturition at term or preterm. Once labor is established, the gearwheel is moving with an inertia too powerful to be stopped with existing tocolytic therapies. An efficacious anti-inflammatory intervention, such as rytvela, is essentially a spanner in the works that suppresses the upstream process of inflammatory amplification and thereby slows the turning of the wheel before it reaches top speed. MAPS, modular accumulation of physiological systems; rytvela, IL-1R allosteric antagonist.

Evolution seems to have prepared humans for the contractile events of labor and the immune effects of giving birth within a single interlaced system. The MAPS (Mitchell and Taggart, 2009), inflammatory load (Talati et al., 2017), and inflammatory burden (Keelan, 2018) concepts feature many parallels, essentially illustrating a single unified theory with different terminology. Instead of an individual trigger initiating birth, what we instead see are a series of increasing interactions involving both contractile and immune pathways. We propose that pro-inflammatory amplification propelled by cooperative interactions between ligands, cells, and tissues presented in this review is an intrinsic part of this accumulative process, driving the increase in inflammatory load or burden in late gestation until reaching that critical mass endpoint resulting in functional progesterone withdrawal and labor induction. Our conceptual framework in **Figure 2** incorporates parts of each of these concepts while focusing in on the paracrine interactions driving inflammatory amplification.

One could depict the processes of human labor as being synonymous with a series of small gears together turning one large wheel. The small components begin to move and then pick up speed, moving more quickly as upregulation continues to be amplified through intrinsic and extrinsic inflammatory interplay. Both fetal membrane senescence and uterine stretch contribute to this escalating pro-inflammatory profile. As these gears turn, their motion affects neighboring gears and initiates movement in the next grouping of wheels. This interaction represents synergy between adjacent tissues. As layer upon layer of gears turn, the speed increases and it becomes more and more difficult to stop the momentum. The cooperativity and amplification depicted in this analogy reveals why we continue to fail at preventing preterm birth in humans, even when we succeed in animal models. This is not a sequence of events, but a series of parallel events occurring concurrently and gradually accumulating over time. We must slow the motion of this wheel before it reaches ‘the point of no return’ and its momentum becomes too strong to suppress. Tocolytic therapies target a wheel already rotating at top speeds and attempt to slow it down, resulting in temporary delay (or no delay at all) until other parallel pathways compensate for the one that is suppressed.

## Changing the PTB Treatment Paradigm

Preterm birth (PTB), defined as birth before the 37^th^ completed week of gestation, affects 5-18% of births in 184 countries worldwide resulting in 15 million babies born preterm each year (March of Dimes et al., 2012). Of those, 1.1 million do not survive (Blencowe et al., 2012; March of Dimes et al., 2012). Those who do survive may face a spectrum of lifelong disabilities, including cerebral palsy, intellectual impairment, chronic lung disease, vision and hearing loss (Committee on Understanding Premature Birth and Assuring Healthy Outcomes, 2007). Globally, PTB complications are the leading cause of newborn death and the second greatest cause of death in children (March of Dimes et al., 2012). In spite of all of the medical advancements of the era and the increasing success of neonatologists in saving babies born at earlier and earlier gestational dates, we still do not have an effective method of prevention for this global problem. Although we have seen progress in survival rates of preterm infants, no intervention advancements have been made, resulting in escalating healthcare costs (Olson et al., 2008).

The strategic approaches for treating PTB have changed over time. Forty years ago, the strategy was to try to completely stop PTB by antagonizing contractile mediators that stimulated myometrial contractions. Drugs in this category included beta-adrenergic agonists, calcium channel blockers or competitors, oxytocin receptor antagonists or prostaglandin synthesis inhibitors (Olson et al., 2008). None of these were very effective as the primary endpoints were to stop contractions with the anticipation the pregnancy would then continue to term. However, this approach failed to consider that the underlying mechanism driving uterine contractions, the fact that the uterus was already activated for labour and therefore unable to revert back to its pre-activation, pregnant state, was not considered. This is why these drugs failed. A modern variation on this concept is that while contractions cannot be permanently stopped, they can be suspended for up to five days. With a 48 hour window, synthetic glucocorticoids can be administered to the mother with enough time to permit them to increase fetal lung surfactant synthesis and release to reduce the incidence or severity of respiratory distress syndrome. This is the strategy employed by ObsEva and its product OBE022, a competitive PGF_2α_ receptor antagonist, which in combination with atosiban diminished delivery at 48 hours 55% more effectively than atosiban alone (www.obseva.com/obe022/). A third contemporary strategy is to intervene farther upstream in the birth cascade at a point that occurs prior to uterine activation in order to prevent the physiological change in the uterus from a pregnant to a parturient state. This is the strategy behind inflammatory antagonists such as rytvela, BSCIs or naloxone.

As it has become clearer that inflammation plays a central role in the delivery process, PTB researchers have turned their attention to the possibility of anti-inflammatory therapies. As the earliest event of the inflammatory birth cascade is the activation of TLRs by DAMPs or PAMPs, the inhibition of specific TLRs has been pursued as a target to suppress amplification. Wildtype mice treated with (+)-naloxone, a TLR4 antagonist, and Tlr4-null mice (Tlr4-/-) both deliver late due to delayed inflammatory upregulation and UAP induction (Wahid et al. 2015). In mouse models of PTB induced by LPS or E.coli, (+)-naloxone successfully delayed PTB, improved birth weights, and reduced the incidence of postnatal death (Chin et al., 2016). Another compound targeting upstream, the broad-spectrum chemokine inhibitor, BSCI, binds the type 2 somatostatin receptor. Inhibition of the type 2 somatostatin receptor results in suppression of a series of chemokines, but the exact mechanism of how BSCI acts on chemokines remains unknown. BSCI delayed LPS-induced PTB in mice and decreased uterine inflammatory expression (Shynlova et al., 2014). In a non-human primate model of Group B Streptococcus-induced PTB, BSCI effectively blocked myometrial contractility and preterm labor, but increased microbial invasion of the amniotic cavity resulting in more severe fetal inflammation and injury (Coleman et al., 2020). These compounds must be further explored, as the broader the inhibition, the greater the potential risk for possible unintended adverse consequences for mother and child. Inhibition of these targets could also possibly suppress important components of the maternal innate immune system, the body’s first line of defense against foreign pathogens. 

Uterine inflammatory amplification comprises different tissue and cell types which, amongst other mediators, involves cytokines IL-1β and IL-6. IL-1β and IL-6 are positioned below TLRs on the birth cascade, but still upstream from current tocolytics. Due to the extent of paracrine interaction between these pathways, suppression of certain actions of IL-1β or IL-6 may be sufficient to suppress amplification before reaching the ‘critical mass’ endpoint or ‘inflammatory load threshold.’ An optimal therapeutic candidate for preterm birth would inhibit both pro-inflammatory and pro-contractile systems. IL-1β and IL-6 represent points of convergence of these systems in the parturition process. One of the controversial issues regarding administration of tocolytics is their use when maternal inflammation or possibly infection is present. In this situation is it better for the fetus to be delivered or not? Some insight can be derived from mouse studies where pro-inflammatory agents (IL-1β, LPS, LTA) were used to stimulate maternal inflammation and preterm labor. Rytvela or 101.10, an IL-1R1 allosteric modulator, decreased the levels of maternal cytokines, blocked the preterm delivery and protected the fetuses from inflammatory harm more effectively than IL-1R antagonist Kineret (Nadeau-Vallée et al., 2015; Nadeau-Vallée et al., 2017). Instead of blocking all signaling from the IL-1R1 receptor like the actions of orthosteric antagonist Kineret, Rytvela is highly specific and selective. Rytvela suppresses signaling through the JNK/AP-1 pathway without abolishing NFκB signaling involved in cytoprotection and immune vigilance. Inflammatory pathways can provoke harmful effects when overexpressed, but they are also involved in crucial signaling that is essential for normal functioning of the human body. We suggest that selective allosteric modulation of IL-1β or IL-6 will increase specificity while minimizing risk of unintended adverse consequences. If inflammatory amplification can be described as a series of gears together propelling one large wheel, a specific and selective inflammatory antagonist such as Rytvela could fill the role of throwing a spanner into the works, slowing the momentum of the large wheel before it reaches top speeds (**Figure 2**).

## Conclusion

In this review, we present cooperativity between ligands, cells, and tissues as a hallmark of human parturition. These studies indicate, as others have proposed through the MAPS and inflammatory load hypotheses, that the accumulation of an increasing number of stimulatory interactions are involved in transitioning the state of pregnancy to the state of parturition. Mitchell and Taggart in reference to human parturition wrote, “Rather than seeking the trigger, experiments need to be designed to investigate synergistic interactions among a variety of physiological systems and tissues” (Mitchell and Taggart, 2009). Further work is required to define the specific inflammatory interactions central to term or preterm labor. For now, it is clear that inflammatory amplification due to localized paracrine interactions in the intrauterine space is an essential component of uterine transition for labor, and IL-1β and IL-6 act as key upstream drivers in this process. This provides a truly potent target for the development of therapies and diagnostic tests to assess when the physiology of labor is approaching term or preterm.

## Author Contributions

KL prepared the manuscript with inputs from WX, MP, SC, and DO. All authors contributed to the article and approved the submitted version.

## Funding

KL is supported by the generosity of the Stollery Children’s Hospital Foundation and supporters of the Alberta Women’s Health Foundation through the Women and Children’s Health Research Institute. The authors are also supported by the Canadian Institutes of Health Research #168858.

## Conflict of Interest

DO and SC are founders of Maternica Therapeutics, Inc.

The remaining authors declare that the research was conducted in the absence of any commercial or financial relationships that could be construed as a potential conflict of interest.

## Publisher’s Note

All claims expressed in this article are solely those of the authors and do not necessarily represent those of their affiliated organizations, or those of the publisher, the editors and the reviewers. Any product that may be evaluated in this article, or claim that may be made by its manufacturer, is not guaranteed or endorsed by the publisher.
